# Effects of gamma frequency binaural beats on attention and anxiety

**DOI:** 10.1007/s12144-023-04681-3

**Published:** 2023-05-04

**Authors:** Natalya Marie Leistiko, Louay Madanat, Wing Kwan Antonia Yeung, James M. Stone

**Affiliations:** 1 King’s College London Institute of Psychiatry, Psychology, and Neuroscience, London, UK; 2 Brighton and Sussex Medical School, University of Sussex, Brighton, UK; 3 Sussex Partnership NHS Foundation Trust, Eastbourne, BN21 2UD UK

**Keywords:** Attention, Anxiety, Binaural Beats, Cognition

## Abstract

Binaural beats (BB) are an auditory phenomenon produced from a combination of two sine waves with slightly different frequencies presented to each ear. Previous research has implicated the role of BBs through brainwave entrainment in potentially giving rise to benefits ranging from enhanced memory and attention to reduced anxiety and stress. Here, we investigated the effect of gamma (40-Hz) BBs on attention using the attention network test (ANT), a previously unused task that assesses three subtypes of attention: Alerting, Orienting, and Executive Control.

Fifty-eight healthy adults performed the ANT remotely under the exposure of 340-Hz BBs and a 380-Hz control tone. All completed a rating scale for levels of anxiety before and after each exposure. Performance on the ANT task (reaction time and error rates) between BB and control groups was evaluated using Wilcoxon signed-rank tests.

We found no significant differences in Reaction Time (RT), Error Rate (ER), or the efficacy of the Attention Networks (AN) between the experimental and control conditions (p > 0.05). We found no effect of BB on self-rated measures of anxiety. Our findings do not provide evidence for improvement in attention with gamma BB.

**Supplementary Information:**

The online version contains supplementary material available at 10.1007/s12144-023-04681-3.

## Introduction

The phenomenon of “binaural beats” (BBs) has gained interest in popular culture recently as a potential method of non-invasive brain stimulation. Alternative medicine practitioners have been promoting ‘binaural beat therapy’ with a range of claimed benefits, going beyond cognitive enhancement to include reduction in stress and anxiety, enhanced meditative states, and even analgesia (Smith, 2019). A small number of scientific studies of BBs have attempted to assess the validity of these claims with mixed results and inconsistent methodologies (Garcia-Argibay et al., 2019).

BBs are a psychophysiological effect which arises when tones with differing frequencies are presented independently to each ear. This results in a third, unified percept in the form of a ‘beat’, formed from the interference pattern between the two tones and arising entirely within the experiencer's mind (Oster, 1973; Wernick & Starr, 1968). This process of sound unification is known in psychoacoustics as binaural integration and is an essential component of sound localisation (Lentz et al., 2014).

Binaural beats can differ in terms of carrier frequency, beat rate, the volume of the carrier tones, and whether or not there are additional (non-binaural) tones presented, all of which affect the perceived salience of the beat (Grose et al., 2012). The BB rate refers to the frequency of the unified BB percept, as determined by the degree of frequency difference between the two tones. This frequency difference, or beat rate, seems to have the largest impact on the type of effect elicited by the BB.

According to one theory supported by several studies that measured electroencephalographic (EEG) activity in response to BBs, the effect occurs through the synchronisation of the frequency of neural oscillations with the frequency of the auditory beat, thus changing the relative power of different EEG frequency ranges (Karino et al., 2004, 2006; Orozco Perez et al., 2020). This effect is known as brainwave entrainment (Huang & Charyton, 2008; Karino et al., 2004; Schwarz & Taylor, 2005). Therefore, since different encephalographic ranges correspond to different cognitive functions, it is hypothesised that BBs can elicit different effects according to the beat frequency that would determine the encephalographic range to be modulated, or ‘entrained’.

The mechanism by which BBs may induce their effect on brain state is still under debate. While some studies have found evidence that BBs lead to entrainment of the brain to audio (Batra et al., 1986; Crespo et al., 2013; Gao et al., 2014; López-Caballero & Escera, 2017; Reedijk et al., 2013; Vernon et al., 2014), this has not been a consistent finding (López-Caballero & Escera, 2017; Shekar et al., 2018; Wahbeh et al., 2007). Another group found that binaural beats of both theta and alpha frequencies increased alpha frequency interhemispheric coherence, which refers to the synchronisation between neural oscillations of the two hemispheres of the brain. They suggested this effect may be due to “binaural integration” rather than entrainment (Solcà et al., 2016).

A number of studies have looked at BBs corresponding to EEG band frequencies (Priyanka et al., 2016; Sih & Tang, 2013); these range from delta (less than 4 Hz) to gamma (greater than 30 Hz) (Rosenberg et al., 2017; Uttal, 2014). Higher frequencies, such as beta and gamma oscillations, are often suggested to be associated with attention (Shekar et al., 2018). While several studies have used 40-Hz BBs and found statistically significant effects on cognitive processes such as attention and memory (Engelbregt et al., 2019; Ross & Lopez, 2020), the evidence is still inconsistent (Crespo et al., 2013). The potential of BBs to improve attention is of particular interest in those with attention deficit disorder or in tasks requiring full attentional concentration (Ross & Lopez, 2020; Engelbregt et al., 2019; Colzato et al., 2017; Hommel et al., 2016).

In this study, we planned to study the effect of binaural beats on attentional networks as described by Posner and Petersen (1990). According to this theory, the attention system utilises three discrete anatomical networks, each responsible for a different set of processes within attention. The alerting network involves brain stem arousal systems and is responsible for switching from a resting state to a state of preparation for responding to an expected signal. The orienting network mainly involves the parietal cortex and facilitates the prioritisation of sensory input by selecting specific information such as locations. Lastly, the executive network mainly involves the cingulate cortex and controls conflict resolution within responses (Petersen & Posner, 2012).

**Fig. 1:**
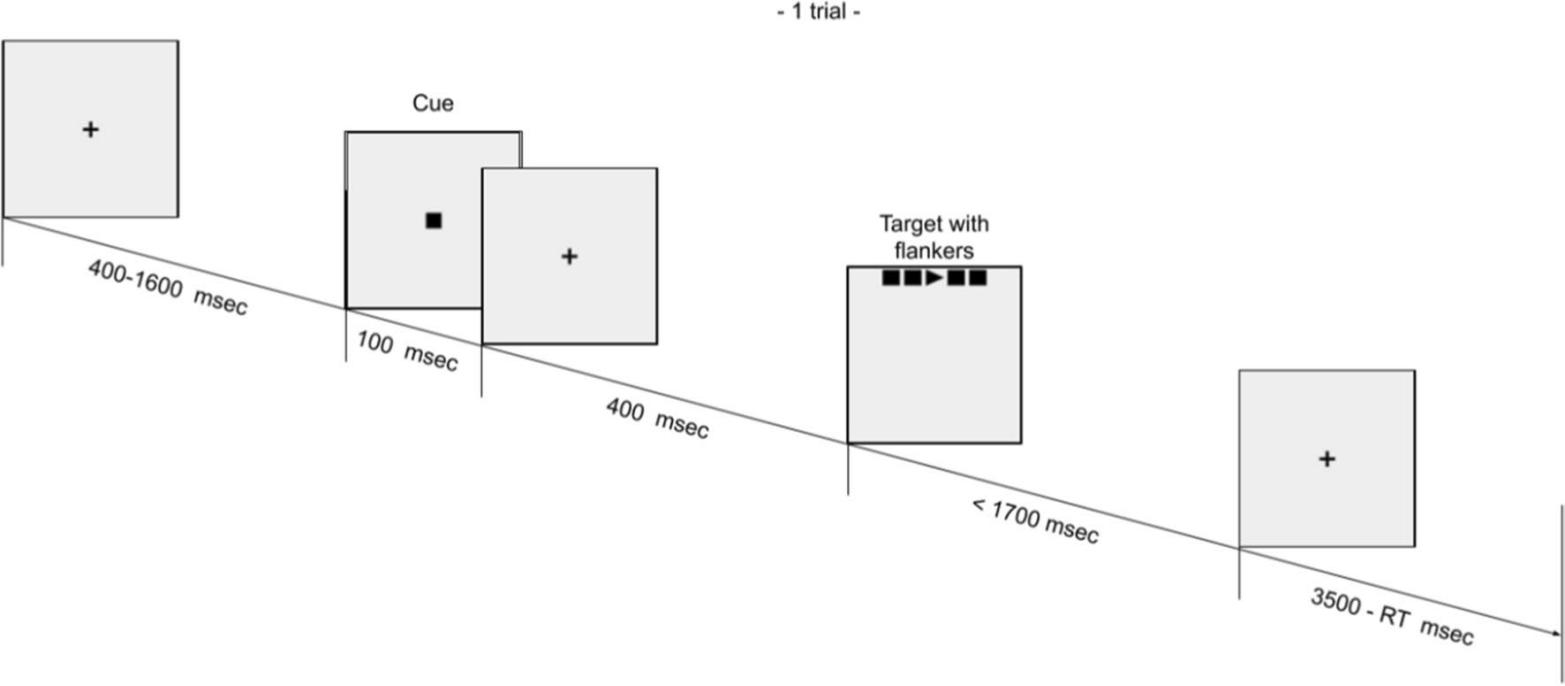
Timeline for 1 trial of the Attention Network Test

The Attention Network Test (ANT) was developed by Fan et al. (2002) as a means to test the efficiency of the three networks of attention. The task consists of a series of trials in which participants respond to the direction of a target arrow that is surrounded by various types of flankers which may be congruent, incongruent, or neutral. In addition, the target may or may not be preceded by various types of cues which may provide temporal or spatial information about the target ahead of its presentation (Fig. 1). By comparing the reaction time (RT) of specific combinations of flanker or cue types, the change in the efficiency of each attentional network from one condition to another can be measured.

The primary aim of this study was to directly test the hypothesis that 40-Hz frequency improves attention during the ANT task (Fan et al., 2002). We also wished to investigate the effect of 40 Hz BBs on the different attentional networks. Functional imaging studies have demonstrated that each of the attentional networks is associated with specific brain regions and neurotransmitters involving attention (Fan et al., 2003, 2005; Pessoa et al., 2002). This approach thus allows a more precise look into the processes implicated in the BB effect, which would then better guide future research in the selection of methodologies and interpretation. It is possible that some of the inconsistent results from previous studies may have occurred due to a lack of consideration of the multimodal nature of attention (Jiang et al., 2011). Using a task such as the ANT that primarily focuses on one cognitive process (i.e. attention) rather than one that involves multiple processes (for example, the digit span task which involves memory in addition to attention) may allow for potential effects to be more reliably isolated to the one process.

Perhaps due to the complex and under-researched nature of this field, existing studies have significant variation in experimental variables such as sample size, frequencies used, timing and length of exposure (Garcia-Argibay et al., 2019). In some instances, fundamental variables such as frequency were not reported at all. In this study, we chose to examine the effects of frequency band 40-Hz because of its association with attention regulation (Reedijik et al., 2015; Garcia-Argibay et al., 2019; Keizer et al., 2010; Ross et al., 2014; Reedijk et al., 2013; Hillebrand et al., 2012).

In a review of 35 previous studies focusing on attention, an average effect size of 0.58 was found (Garcia-Argibay et al., 2019). Using this estimate of effect size, we conducted a power analysis and found that a sample size of at least 34 participants was necessary in order to observe an effect at the level of *p* < 0.05 with a power of 0.95.

## Materials and Methods

### Participants

Sixty healthy, adult participants (30 males, 30 females) were initially recruited by email or across social networks; two were excluded due to technical reasons (therefore, *N* = 58). The participants were located in multiple geographical locations: United States (California and New Jersey), United Kingdom (London), Jordan, and Hong Kong. Ages ranged from 18 to 62 years (M = 26, SD = 10). The participants’ education levels were from high school to postgraduate. All participants had normal or corrected-to-normal sight. Exclusion criteria included deafness in one or both ears and a history of epilepsy. Those who had extensive experience with BBs and sufficient knowledge in sound production were also excluded to maintain the integrity of the control condition, as prior knowledge may potentially lead to preconceived notions on the efficacy of BBs. All mentions of BBs were hidden from participants throughout the study, instead using the term ‘auditory tones’. Signed informed consents were collected prior to the experiment. All participants were financially compensated with a five-pound or equivalent Amazon voucher.

### Ethics Statement

The protocol and monetary compensation were approved by the local ethical committee (King’s College London, Institute of Psychiatry, Psychology & Neuroscience). All procedures were performed in accordance with the ethical standards. The study was reviewed and approved by King’s College London Research Ethics Committee.

### Experiment

The experiment was designed in PsychoPy and JavaScript and conducted online via Pavlovia.org, a hosting platform that allows researchers to run and share experiments online. A version of the attention network test available on pavlovia.com, which followed the original Fan et al. (2002) design, was modified for the purposes of this study.

### Attention Network Test

The ANT was designed to assess the three aspects of attention (Posner, 1980) by combining a cued RT test (Posner, 1980) and a flanker test (Eriksen & Eriksen, 1974). All participants performed an initial small practice block of 10 trials, receiving feedback regarding their speed and accuracy. This was without audio, to minimise practice effects and to familiarise themselves with the procedure. The main portion of the test is split into three blocks of 96 trials each, with a total of 576 trials for both runs of the test. In each trial, participants were presented one of four cues and three flanker-surrounded targets and were required to determine the direction of the central target arrow, left or right (Fig. 1). The four cue conditions were “blank”, “both”, “centre”, and “spatial” and three flankers were “congruent”, “incongruent”, and “neutral”, as in Fan et al., 2002. Responses were made using the arrow keys on a QWERTY keyboard.

### Binaural Beat Stimuli

**Fig. 2:**
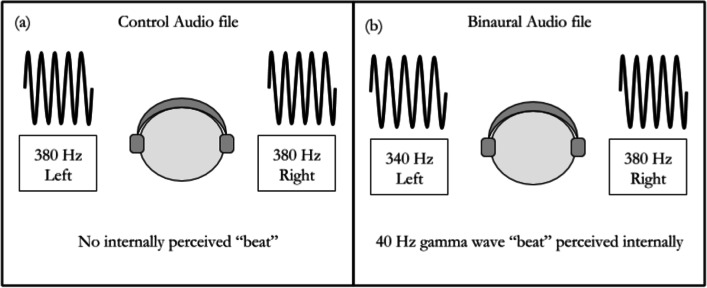
Difference between Control and Binaural Audio file structures

The BB file consisted of a 340 Hz wave on the left side and a 380 Hz wave on the right, creating a 40 Hz BB (Fig. 2). The control condition audio file was a 340 Hz stereo tone, in line with previous similar studies (Colzato et al., 2017; Hommel et al., 2016). Both audio files were generated using synthesisers in Ableton Live 10, masked with pink noise and verified using a stock digital tuner at an accuracy of one decimal point. Pink noise, which has attenuated higher frequencies compared to white noise, was used instead because it is found in numerous natural settings, especially in human systems (Acosta et al., 2019; Benjafield, 2017; Halley, 1996).

### Mental States Questionnaire

A Mental States questionnaire was created to determine the change in mental states or emotions between the control audio and the BB audio. Participants were instructed to rate the following from a scale of 0 to 100: happiness (0 = sad/low, 100 = happy), alertness (0 = exhausted, 100 = energetic), anxiety (0 = relaxed, 100 = most anxious), and clear-headedness (0 = dizzy/disoriented, 100 = clear-headed).

### Procedure

All experiment components were run by the participants: a precise timeline and set of instructions for experiment day procedure and room set-up were sent to the participant at least 24 h before the assigned time slot (see supplemental information). Headphones or earphones were confirmed to produce stereo sound to fully allow for BBs to be heard. Sound volume calibration measures were done using an online audiogram hearing test to aim towards controlled audio exposure (AudioCheck.net, n.d.). Testing took place at the participant’s home in a quiet, dim-lit room to minimise distractions. Participants were asked to abstain from alcohol, drugs, nicotine, and caffeine for 24 h prior to the study, unless it is part of their routine.

On the day of the experiment, participants were refreshed on the timeline of the experiment and were instructed on how to set up their surrounding environment via virtual communication platforms (e.g., Zoom). Each participant was connected with their respective host to confirm that they were following the instructions and to answer any questions they had during the experiment, with the host otherwise staying muted.

Participants were exposed to the audio stimulus for five minutes at the start of each run before starting the test. This continued during the test. The participants were randomly assigned to the two experimental audio order conditions: control-first or binaural-first, modelled after Garcia-Argibay et al. (2019). During the experiment, before and after each run (audio file), each participant filled out the MSQ with separate forms for each audio file (see supplemental information). Between the runs, participants were required to take a 3–5 min mandatory break.

### Data Collection and Analysis

Data was collected from Pavlovia and analysed with Jamovi. Any RT data that exceeded four standard deviations (SD) above or below each individual participant’s mean was excluded from all analyses; 2 participants exceeded 4 SD and were thus excluded (final *N* = 58).

We analysed total RT and ER data using Wilcoxon paired-samples. The AN scores were determined by the following formulas: alerting = (average RT of blank-cue – average RT of both-cue), orienting = (average RT of centre-cue – average RT of spatial-cue), and executive function = (average RT of incongruent-flanker – average RT of congruent-flanker) (Shekar et al., 2018). We analysed the effect of cue type and flanker type on ER was using ANOVA. We analysed change in mental state measures between control audio and BB using Wilcoxon paired-samples.

## Results

### Reaction Time

There was no effect of BBs vs. control audio on RT (*Z* = 958, *p* = 0.43; Table 1).

**Table 1:** Reaction Time for Audio Files (msec)

Condition	Mean	Median	SD
Control	547	542	83
Binaural	545	519	88

### Error Rate

There was no effect of BBs vs. control audio on ER (*Z* = 626, *p* = 0.16). Cue type [F(2.47, 141) = 4.69, *p* < 0.006] and flanker type [F(1.02, 58.0) = 48.7, *p* < 0.01] were both found to have a significant effect on ER. There was also an interaction between cue and flanker [F(3.03, 173) = 8.61, *p* < 0.01]. Mean ER decreased when spatial cues directed the participants’ eyes to the location of the upcoming target and when congruent flankers surrounded the target arrow (Table 2).

**Table 2:** Error Rate for Audio File (%)

Condition	Mean	Median	SD
Control	2.93	1.93	2.65
Binaural	3.90	2.50	5.20

### Attention Networks

There were no significant effects found for BB vs. control audio for any AN: Alerting [*Z* = 673, *p* = 0.159], Orientation [*Z* = 1091, *p* = 0.069], Executive Function [*Z* = 944, *p* = 0.496] (Table 3).

**Table 3:** Change in Attention Network efficacy for Audio Files (msec)

Condition	AN	Mean	Median	SD
Control	Alerting	27.3	29.1	18.5
	Orienting	23.1	18.9	22.5
	Executive Function	121	121	32.3
Binaural	Alerting	31.5	34.3	20.3
	Orienting	17.6	16.2	17.2
	Executive Function	116	114	37.1

### Mental State

There was no significant difference in change in reported mental state for BB vs. control audio: anxiety [*Z* = 593, *p* = 0.399], happiness [*Z* = 492, *p* = 0.614], alertness [*Z* = 792, *p* = 0.137], clear-headedness [*Z* = 396, *p* = 0.249].

## Discussion

The main aim of this study was to investigate the effect of BBs on the efficiency of the three attentional networks by analysing the performance of 58 participants on a modified within-subjects design of the ANT task. Results yielded no overall effects of BBs on performance on the ANT task, neither by RT nor ER. Further analysis of the effect on individual network efficiencies similarly yielded non-significant results.

The results of the analyses of variance for cue and flanker types corresponded with the original ANT design as expected (Fan et al., 2002). The absence of a cue slowed RT slightly, regardless of other cue types. This reflects the benefit of the additional temporal and potentially spatial information that cues provide regarding onset of the targets. Spatial cues provided the fastest RT among cue types due to their combined contribution of temporal and spatial information about the target stimuli. However, this difference was to a lesser extent than that between the altogether absence and presence of a cue, suggesting an overall larger alerting effect on RT compared to an orienting effect.

The effect of incongruence was also in line with the original study as the most significant effect on both RT and ER, increasing each substantially compared to other conditions. In fact, no other condition had a significant effect on ER besides incongruence. This suggests that the errors were largely caused by conflicting information, rather than deficiencies in alerting or orienting networks.

The speed-accuracy trade-off (SAT) states that the mean RT for correct answers will generally be longer than the mean RT for incorrect answers (Heitz, 2014; Heitz & Schall, 2012). At the same time, the probability of making a correct decision increases as information is learned but learning all the necessary information takes time and can lead to slower RTs (Wang et al., 2002). SAT and the idea of channel enhancement, (Bogacz et al., 2010; le Scouarnec et al., 2001; Reed, 1973) could also be used to explain why ER decreases as a product of RT, cue, and flanker. Only flanker type and the interaction of cue and flanker were found to be significant in effect for ER, as supposed to cue and flanker separately for RT. Each type of cue either draws the eyes closer or further from the coming flanked target, but that acts as more of a preparation for the upcoming decision. The flankers cause the participants to spend time discerning the direction of the target, which can affect RT, but is more important to ER. The “incongruent” and “neutral” flankers hinder and distract the participant from the target while “congruent” flankers aid the eyes in figuring out the direction of the target.

Due to COVID-19 regulations, the study design had to be modified to allow for it to be conducted remotely, which led to several implications. A variety of factors could not be controlled as well as if the study was performed in a centralised, controlled lab environment, such as: headphones used, sound volume, background noise, distractions, or technical issues.

Conducting the study remotely nevertheless also had its benefits. Our ability to obtain a sufficiently large sample size (*n* = 58) was facilitated partly due to the removal of geographical restrictions or transportation requirements in recruitment. This geographical freedom also allowed for participants from all over the world to participate, rather than primarily students in a local area.

Another strength not always found in similar studies was the within-subjects design of the study: this provided a higher statistical power than a between-subjects design, which can have an influence on the sample size (Schäfer & Schwarz, 2019) By ensuring that each participant was tested on both conditions in succession, the effect of interindividual differences such as age and experience would also have been minimised. Finally, we believe this study’s methodology is built on a solid rationale, which in turn can allow future studies to build upon it in a more robust fashion with potentially more conclusive results, as discussed below.

Further research should take into consideration the limitations faced in this study and focus on addressing the same questions in a controlled environment. This study investigated the effect of BB for a single exposure for approximately 40 min. Higher exposure times have been linked to larger effect size (Garcia-Argibay et al., 2019), though previous studies have refuted this claim (Kennel et al., 2010). Another consideration would be masking the audio files with other “colours” of sound (i.e. white, pink, or brown noise) (Halley, 1996), to determine whether the masking noise affects any aspect of the experiment. It may also be of interest to expand the question of BBs’ effect on attention to the entrainment of the various brainwave states (Huang & Charyton, 2008), other cognitive functions like sleep (Fan et al., 2002; Gamboz et al., 2010) or complex tasks such as driving (Weaver et al., 2009), listening to, or playing music (Jiang et al., 2011).

## Conclusion

In summary, we found that exposure to gamma BBs did not yield significant changes in the assessment of the efficacy of attentional networks. The methods used in this provide a framework to study attention using different BB frequencies.

## Supplementary Information

Below is the link to the electronic supplementary material.

Supplementary file1 (PNG 155 KB)

Supplementary file2 (PNG 80 KB)

## Data Availability

Data are available through contact with the corresponding author.
