# Supplementary figures and images for: Effects of gamma frequency binaural beats on attention and anxiety
Source: Curr Psychol. 2023 May 4:1–8. Online ahead of print. doi: 10.1007/s12144-023-04681-3 (PMC10157589; doi:10.1007/s12144-023-04681-3)

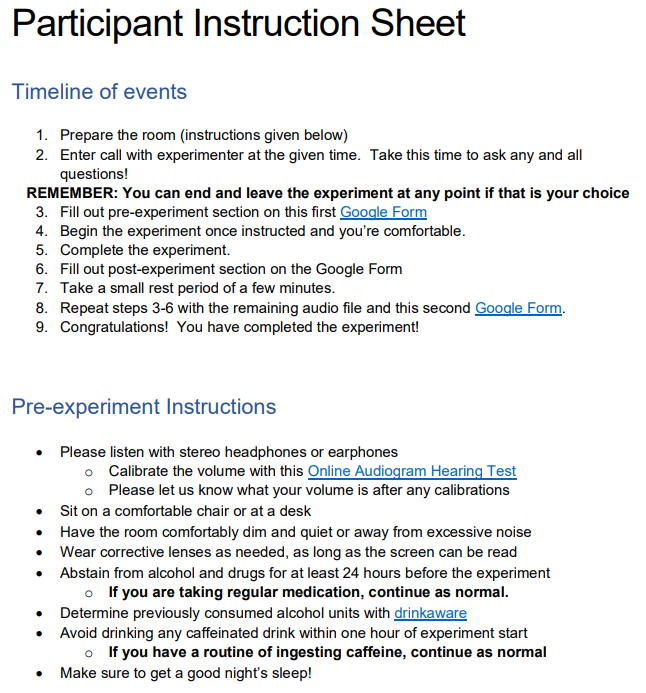

Supplement: Supplementary file 1 — Supplementary file1 (PNG 155 KB) [file 12144_2023_4681_MOESM1_ESM.png]

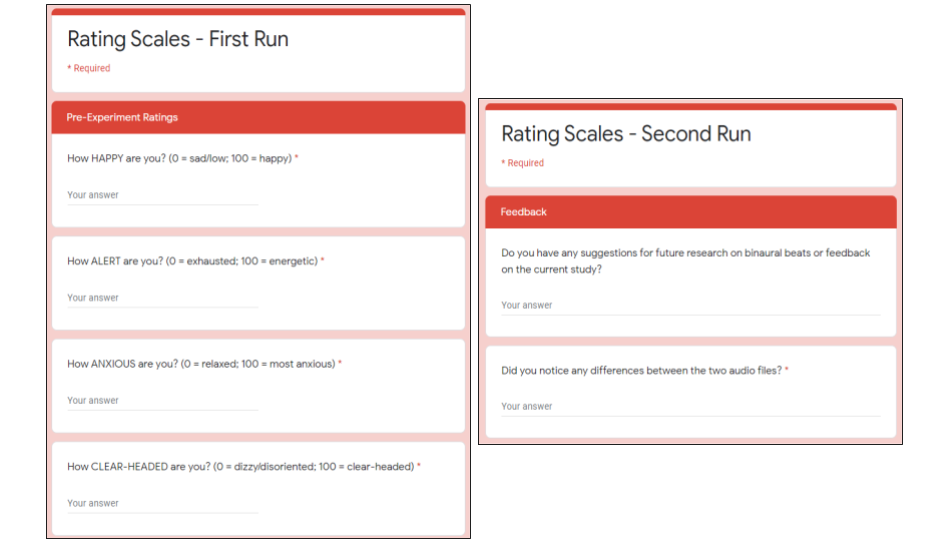

Supplement: Supplementary file 2 — Supplementary file2 (PNG 80 KB) [file 12144_2023_4681_MOESM2_ESM.png]
